# Genetic determinants for methotrexate response in juvenile idiopathic arthritis

**DOI:** 10.3389/fphar.2015.00052

**Published:** 2015-03-23

**Authors:** Serena Pastore, Gabriele Stocco, Diego Favretto, Sara De Iudicibus, Andrea Taddio, Pio d’Adamo, Noelia Malusà, Riccardo Addobbati, Giuliana Decorti, Loredana Lepore, Alessandro Ventura

**Affiliations:** ^1^Institute for Maternal and Child Health IRCCS Burlo Garofolo, TriesteItaly; ^2^Department of Life Sciences, University of Trieste, TriesteItaly; ^3^Department of Medical, Surgical and Health Sciences, University of Trieste, TriesteItaly; ^4^Department of Prevention, Azienda Servizi Sanitari 1, TriesteItaly

**Keywords:** juvenile idiopathic arthritis, methotrexate, pharmacogenomics, genome-wide association study, gene expression profiling

## Abstract

Juvenile idiopathic arthritis (JIAs) is the most common chronic rheumatic disease of childhood and is an important cause of disability. The folic acid analog methotrexate is the first choice disease-modifying anti-rheumatic drug in this disease, however, 35–45% of patients fail to respond. Molecular elements, such as variants in genes of pharmacological relevance, influencing response to methotrexate in JIA, would be important to individualize treatment strategies. Several studies have evaluated the effects of candidate genetic variants in the complex pathway of genes involved in methotrexate pharmacodynamics and pharmacokinetics, however, results are still contrasting and no definitive genetic marker of methotrexate response useful for the clinician to tailor therapy of children with JIA has been identified. Recently, genome-wide approaches have been applied, identifying new potential biological processes involved in methotrexate response in JIA such as TGF-beta signaling and calcium channels. If these genomic results are properly validated and integrated with innovative analyses comprising deep sequencing, epigenetics, and pharmacokinetics, they will greatly contribute to personalize therapy with methotrexate in children with JIA.

## Introduction: Juvenile Idiopathic Arthritis and Methotrexate

Juvenile idiopathic arthritis (JIA) is the most common chronic rheumatic disease of childhood and is an important cause of disability (Ravelli and Martini, 2007). A rapid control of inflammation allows avoiding structural damage and growth impairment. Today there are lots of treatment options, especially biologics targeting cytokines are continuously developing (Lovell et al., 2000, 2008; Brunner et al., 2014). Among the conventional disease modifying anti-rheumatic drugs (DMARDs), methotrexate remains first-line therapy for children with JIA, due to long-term efficacy, safety profile and low-cost (Giannini et al., 1992; Ramanan et al., 2003). It is used as first disease-modifying and steroid-sparing medication, but it is useful also in synergism with biological drugs to prevent antibody formation (Lovell et al., 2008; Horneff et al., 2009).

Recent improvements in treatment and management of JIA have increased expectations from clinicians, parents, and patients for complete and early control of disease. Pharmacological determinants, including genetic markers, useful to predict treatment outcome, may be crucial to select the right drug for each patient, maximizing treatment efficacy, and reducing long-term complications of the disease (Stocco et al., 2012). However, research is still ongoing to identify the appropriate markers for methotrexate and the other DMARDs (Cobb et al., 2014; Schmeling et al., 2014).

**FIGURE 1:**
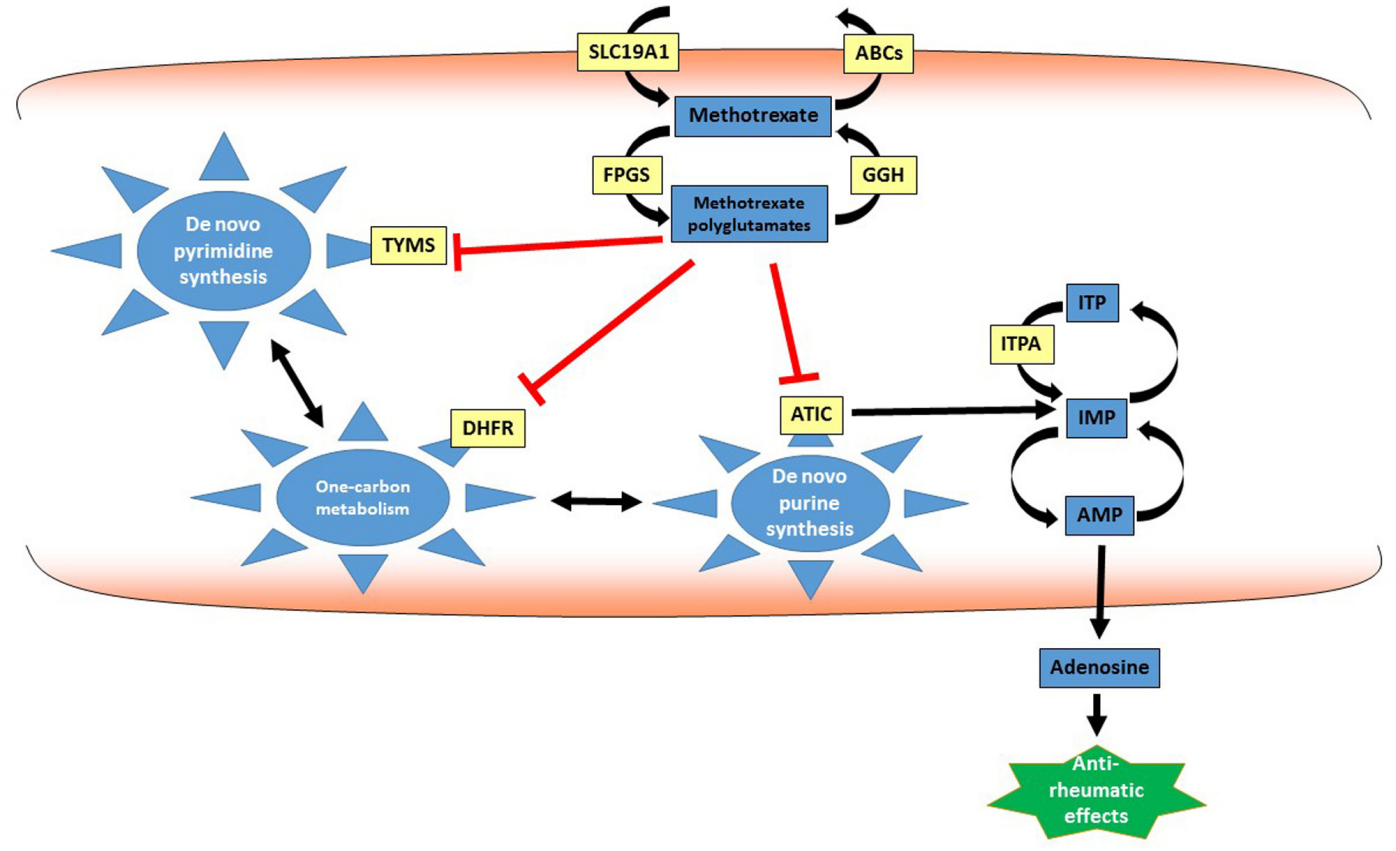
**Methotrexate pathway.** Methotrexate and its active polyglutamate metabolites interfere with several intracellular metabolic processes in particular one-carbon metabolism and *de novo* purine and pyrimidine synthesis, by inhibiting the activity of key enzymes such as DHFR, ATIC, and TYMS. This antimetabolic effect may lead to alteration in intracellular nucleotide pools and increased adenosine release, determining the anti-rheumatic effect of methotrexate. ABCs, ATP-binding cassette transporters; AMP, adenosine monophosphate; ATIC, adenosine ribonucleotide transformylase; DHFR, dihydrofolate reductase; FPGS, folylpolyglutamate synthase; GGH, gamma-glutamyl hydrolase; IMP, inosine monophosphate; ITP: inosine triphosphate; ITPA, inosine triphosphate-pyrophosphatase; SLC19A1, solute carrier family 19 member 1; TYMS, thymidylate synthetase.

The folic acid analog methotrexate is the first choice DMARD in JIA (Giannini et al., 1992; Becker, 2011), however, 35–45% of patients fail to respond (Ruperto et al., 2004; Wallace et al., 2012). Methotrexate determines its antiproliferative activity through inhibition of several enzymatic pathways in which folates are relevant, in particular by interfering with the activity of the enzymes dihydrofolate reductase (DHFR) and thymidylate synthetase (TYMS; Mikkelsen et al., 2011). However, the mechanism of action of methotrexate in JIA is still under investigation, and its anti-inflammatory effects may be mediated by additional mechanisms to folate antagonism (Chan and Cronstein, 2010). Indeed, co-treatment with folic acid prevents toxic effects of methotrexate and not the therapeutic effects in JIA (Morgan and Baggott, 2010; Schmeling et al., 2014). Additional mechanisms may involve adenosine release, inhibition of spermine/spermidine production and/or alteration of cellular redox state (Cronstein, 2005). In particular, the efficacy of methotrexate in JIA has been ascribed to the release of anti-inflammatory adenosine, through inhibition of 5-aminoimidazole-4-carboxamide ribonucleotide formyltransferase (ATIC) and consequent accumulation of intracellular purine nucleotides (Cronstein et al., 1991; Dervieux et al., 2011; **Figure 1**).

Identification of patients who are likely to respond to methotrexate would be very important for the clinician. Clinical improvement is indeed usually seen after 6–12 weeks of methotrexate therapy. Given this time lag between methotrexate treatment initiation and the patient response, it would be particularly useful to determine a priori the probability of therapeutic efficacy: the delay in identifying the optimal treatment at an early stage of disease can influence the long-term joint damage. The use of fully validated pharmacogenomics markers may allow to identify responsive patients that could be treated with methotrexate (Schmeling et al., 2014). On the contrary, patients with variants associated with lack of efficacy for methotrexate should be switched more rapidly to a more aggressive treatment (for example methotrexate plus biologics; Pastore et al., 2014). Early identification of the right therapy could prevent deformity and growth disturbance, with great benefit for children with JIA.

## Pharmacogenetics of Methotrexate Response

Several studies have reported the effect of candidate genetic variants on methotrexate sensitivity. These studies demonstrate that genetic variants of enzymes and transporters in the methotrexate pathway, such as methylenetetrahydrofolate reductase (MTHFR), aminoimidazole carboxamide adenosine ribonucleotide transformylase (ATIC) and ATP-binding cassette transporters (ABCs) are associated with methotrexate response in JIA: this topic has been reviewed recently (Schmeling et al., 2014). Our experience on methotrexate pharmacogenetics is related to the evaluation of the most common functional variants in ATIC, inosine triphosphate-pyrophosphatase (ITPA) and solute carrier family 19 member 1 (SLC19A1): we selected these candidate variants on the basis of recent studies evaluating comprehensively genetic polymorphisms in the methotrexate pharmacokinetic and pharmacodynamic pathways in patients with JIA and rheumatoid arthritis (RAs; Dervieux et al., 2011; Hinks et al., 2011; Owen et al., 2012). In our analysis (Pastore et al., 2014), the most common functional variants of ATIC, ITPA, and SLC19A1 were associated with clinical response to methotrexate evaluated as remission stable for a 6-months period, as ACRPed score and as change in Juvenile Arthritis Disease Score (JADAS) in 69 patients with JIA. ATIC rs2372536 GG genotype was associated with improved clinical remission (adjusted *p*-value = 0.0090). For ITPA, rs1127354 A variant was associated with reduced clinical remission (adjusted *p*-value = 0.028). ITPA enzymatic activity, measured in patients’ erythrocytes, showed that also patients with wild-type ITPA, but with low ITPA activity, did not respond to therapy (*p*-value = 0.0024). SLC19A1 rs1051266 variant showed an association with worse response in terms of change in JADAS score after 6 months of therapy (adjusted *p* = 0.036). The associations described in this study about explored genetic variants and different indicators of clinical response are heterogeneous: this may be secondary to the relatively small number of patients recruited. The results, therefore, have to be interpreted as preliminary and need further confirmatory studies on larger cohort of patients.

Different studies, including this one, have therefore evaluated the effects of candidate genetic variants in the complex pathway of genes involved in methotrexate pharmacokinetics and pharmacodynamics on response to methotrexate in children with JIA; however, results are still contrasting and no definitive genetic marker of methotrexate response useful for the clinician to tailor therapy of children with JIA has been identified. This may be due to the lack of studies considering comprehensively all the relevant variants, even with an agnostic genome-wide approach since the mechanism of action of methotrexate in JIA is still poorly understood, or fine mapping of genetic variants in relevant candidate genes in a sufficiently powered and prospectively followed-up patients’ population with standardized protocols, accounting for all non-genetic potential confounders. Therefore, more research is needed to fully validate pharmacogenetic markers for methotrexate response in JIA, considering even the effect of epigenetic features, such as DNA methylation, as discussed later in this review.

## Recent Pharmacogenomic Studies on Methotrexate Response in JIA

Genome-wide approaches, using genome-wide DNA scans and also gene expression arrays or proteomics, can help in understanding the genetic contribution to variation in phenotypes of pharmacological interest, such as response to methotrexate in JIA, by providing a relatively unbiased survey of the genome to identify previously unrecognized relevant genes. Indeed, candidate gene approaches have the potential drawback that they depend on accurate previous knowledge on the molecular mechanisms underpinning the phenotype of interest. Genome-wide studies are then particularly useful if limited knowledge is available on the molecular basis of the phenotype of interest. A limitation of genome-wide approaches is that, given the large number of polymorphisms examined, many statistically significant findings are false positives (type I error). Moreover, many platforms available for genome-wide DNA analysis characterize for each gene just a panel of SNPs, tag SNPs, selected to be representative of multiple SNPs that are in linkage disequilibrium, therefore the causative variants are often not directly typed. The large number of SNPs examined and direct characterization of tag SNPs only, determines, in genome-wide studies, the need for large cohorts of patients to identify truly significant associations.

The first genome-wide study considering genetic variants for methotrexate response in JIA was recently published (Cobb et al., 2014). This study had the overall aim to improve the understanding of the variability in response to methotrexate treatment observed in children with JIA and ultimately define a multifactorial model of response outcomes, involving multiple genetic variants, and environmental factors. The study was performed by the CHARMS-JIA GWAS International Consortium and considered, following stringent quality control for the genomic analysis, 694 JIA cases (from Czech Republic, the Netherlands and UK). Demographic and clinical variables were collected at baseline and after 6 months (median 6.2 months, range 4–8 months) of therapy with methotrexate (median dose 11.3 mg/m^2^, range 10–15). Clinical response was evaluated using the ACRPed criteria with four categories: non-responders (reference category, 31%), ACRPed30 (8.6%), ACRPed50 (14.6%), and ACRPed70 (45.8%); association between genotype and response to methotrexate was analyzed using ordinal regression. Moreover, each of the core-set variables (erythrocyte sedimentation rate, childhood health assessment questionnaire, active joint count (AJC), limited joint count, physician’s global assessment on a visual analog scale and patient global assessment) determining the ACRPed score was analyzed individually, using linear regression, recording the change between baseline, and follow-up. Genotypes were determined on patients’ DNA using Illumina HumanOmniExpress Infinium array, which in this study, after appropriate quality control, allowed genotyping of 586,062 SNPs. In a first phase analysis, some regions were selected by searching for clusters of associated SNPs (*p* < 0.001 in at least two of the seven analyses). In a second phase, analysis of these regions was refined, using SNP imputation to increase density of SNP coverage in these regions (imputation was done against the 1000 Genome Project reference panel of around 37 million SNPs). None of the potential confounding variables tested (gender, ILAR-JIA subtype, center, age at treatment baseline, duration of treatment, time of treatment, steroid treatment, ethnicity) were associated with all six individual core-set variables: therefore no adjustment for these clinical and demographic covariates was done. The first phase analysis identified 31 genetic regions (*p* < 0.001). Phase II analysis, which included additional SNPs in the significant regions, typed by imputation, identified 14 regions fulfilling a more conservative significance threshold (*p* < 1.0 × 10^-5^). The variant rs136855 was the most significant (*p* = 9.81 × 10^-8^) within an intron of the calcium channel CACNA1I, associated with AJC: this gene is implicated in calcium signaling in neurons and may have other roles that yet have to be discovered, related to methotrexate efficacy. Moreover, a significant region included the ABC transporter superfamily member CFTR/ABCC7, important for drug transport and elimination and belonging to the same family of ABCC3, known to be involved in the eﬄux of methotrexate. Finally, two regions (including variants of ZMIZ1 and TGFI1) showed intriguing functional overlap with the immune suppressive factor TGF-beta signaling pathway, important for the balance between Th17 and Treg cells, directly involved in JIA severity, and previously implicated by gene expression profiling as relevant for methotrexate response in JIA (Moncrieffe et al., 2010). Although this interesting two-step approach, the study failed to identify an association at a genome-wide level (5 × 10^-8^) and there is not a replica on an independent cohort (a very common method to reduce false positive associations). A previous gene expression study, performed also by the CHARMS-JIA research group, applied transcriptome analysis to identify novel pathways/genes altered by methotrexate in JIA patients and potentially useful for therapy personalization. Gene expression profiling before and after methotrexate treatment was performed on 11 children with JIA, evaluating response after 6 months of treatment. Genes displaying the most significant changes in gene expression were selected for SNP genotyping. More than 1000 probesets were differentially expressed (fold change >1.7, *p* < 0.05) after methotrexate treatment in these patients; analysis focused on seven patients with full response to methotrexate (ACRPed70), identified 1065 differentially expressed probes; after correction for multiple testing, 87 probe sets in 62 genes resulted as highly differentially expressed. Among these, selection for further analysis by genotyping was done on six genes selected on the basis of the level of fold change and potential biological relevance. Metastasis-associated lung adenocarcinoma transcript 1, upregulated in cells treated by several chemotherapeutic drugs including methotrexate, displayed the highest fold-change (12.8, *p*-value corrected for multiple changes = 0.004) after methotrexate treatment in patients with JIA. Moreover, zinc finger enhancer protein 1 (ZEB1), also known as transcription factor 8 (TCF8), which enhances signaling of TGF-beta was differentially expressed after methotrexate treatment (fold change 5.3, *p*-value = 0.014). The relevance of TGF-beta signaling on methotrexate response was therefore identified by two studies with an agnostic approach, initially considering gene-expression and then by genome-wide SNP analysis (Moncrieffe et al., 2010).

Recently, the Trial of Early Aggressive Therapy (TREAT) in JIA patients, compared 2 therapeutic regimens for initial therapy of newly diagnosed polyarticular JIA, i.e., subcutaneous methotrexate at a relatively high dose (0.5 mg/m^2^/week, max 40 mg/m^2^/week) vs. a combination of subcutaneous methotrexate (same dose as other arm), etanercept and oral prednisolone. The TREAT study also comprised agnostic gene expression studies, to determine whether RNA profiles measured in peripheral whole blood, by Illumina WG-6 v3, or Illumina HT-12 v4 human whole genome microarrays, could be used to determine the therapeutic outcome. Results obtained showed that the prognosis at 6 months in terms of clinically inactive disease was strongly associated with gene expression at presentation, irrespective of treatment with methotrexate alone or with the more intense combination of methotrexate, TNF-alpha inhibitors and glucocorticoids. While the models developed in this study were not statistically robust, given the relatively small number of patients considered (*n* = 44), these data support the existence of a genomic component to prognosis for patients with JIA, that may be independent from treatment with methotrexate or more aggressive combinations. At this stage, the authors do not provide details on genes associated in their study with prognosis: further studies in larger patient’s populations are needed (Jiang et al., 2014).

These genome-wide studies with an agnostic profile suggest that multiple genes determine methotrexate efficacy in JIA, and not only those involved in methotrexate pharmacokinetics and pharmacodynamics. Novel pathways and mechanisms therefore may be relevant to elucidate methotrexate efficacy. If validated by future targeted replication studies, these novel variants could improve previously developed methotrexate efficacy predictions model in JIA. These genome-wide studies did not replicate candidate genes studies, since they characterize the contribution of a different type of genetic variation: indeed, in the genome-wide array used for the SNP analysis, most functional variants, such as the common SNPs in ATIC, ITPA, and SLC19A1 analyzed in our study, are not directly typed and the tag SNPs genotyped may have an indirect effect too diluted to be evident with the sample size considered. The variants identified as significant in this study, however, shed light on previously unrecognized relevant biological mechanisms to predict methotrexate efficacy in JIA.

While there is a paucity of GWAS data on MTX response in JIA, various studies have been published in the context of other subtypes of RAs or other conditions, considering the effect the MTX alone or in association with other medications (Wang et al., 2013; Senapati et al., 2014). In a recent paper, Senapati et al. (2014) reported genome-wide genotyping data for 457 RA patients, 297 good and 160 poor responders on MTX monotherapy. Ten novel suggestive loci were associated with poor response to methotrexate; interestingly, the associations of published candidate genes, namely DHFR (*P* = 0.014), FPGS (*P* = 0.035), and TYMS (*P* = 0.005) and purine and nucleotide metabolism pathways, were confirmed, even if not significant at genome-wide level (Senapati et al., 2014).

## Further Steps

Advancement in sequencing technology now allows deep sequencing of a panel of candidate genes in a reasonable time and with acceptable costs. Moreover the development of specific algorithms (i.e., Polyphen-2, MutationTaster, SIFT) has greatly improved the possibility of evaluating the functional impact of novel genetic variants observed in these studies. Recent studies have shown that evaluation of rare variants is particularly important to properly predict the phenotype of interest, such as methotrexate pharmacokinetics and response (Ramsey et al., 2012): from deep resequencing of methotrexate transporter SLCO1B1 exons in 699 children, the authors identified 93 SNPs, 15 of which were non-synonymous (NS). Three of these NS SNPs were common, with a minor allele frequency (MAF) >5%, one had low frequency (MAF 1%–5%), and 11 were rare (MAF <1%). NS SNPs (common or rare) predicted to be functionally damaging were more likely to be found among patients with the lowest methotrexate clearance than patients with high clearance. SLCO1B1 variants accounted for 10.7% of the population variability in clearance. Of that variability, common NS variants accounted for the majority, but rare damaging NS variants constituted 17.8% of SLCO1B1’s effects (1.9% of total variation) and had larger effect sizes than common NS variants.

Besides evaluation of DNA sequence or mRNA profiles, other elements of genetic variants may be related to drug effects: epigenetic features, such DNA methylation or microRNA expression profiles, influence drug response (Ivanov et al., 2014) and analysis of their effects on clinical response is completely missing in patients with JIA treated with methotrexate.

The concentration of methotrexate polyglutamates (MTX-PG) in patients’ erythrocytes has been proposed as a marker of efficacy of treatment with methotrexate in patients with rheumatologic diseases: levels of MTX-PG metabolites are higher in patients who respond to therapy; however, some studies report conflicting results. Data currently available in the literature on the usefulness of the measurement of MTX-PG metabolites in patients with JIA is limited and the value of these tests in predicting clinical response is still unclear (Stocco et al., 2012). An association between MTX-PG levels and response to therapy has been recently confirmed in the first prospective study in JIA (Ćalasan et al., 2015). One hundred and thirteen JIA patients were followed from methotrexate start until 12 months. Erythrocyte MTX-PG with 1–5 glutamate residues were measured at 3 months with tandem mass spectrometry. The clinical outcomes considered were JADAS and incidence of adverse effects. Median JADAS decreased from 12.7 (inter-quartile range, IQR: 7.8–18.2) at baseline to 2.9 (IQR: 0.1–6.5) at 12 months. Higher concentrations of MTX-PG3, MTX-PG4, MTX-PG5, and MTX-PG3-5 were associated with lower disease activity at 3 months and over 1 year. No significant association was detected between MTX-PG concentration and incidence of adverse effects (methotrexate intolerance, hepatotoxicity and bone marrow suppression). Therefore, erythrocyte MTX-PGs are associated with lower JADAS at 3 months and during 1 year of methotrexate treatment and could be a plausible candidate for therapeutic drug monitoring of methotrexate in JIA. Genomic variants associated with MTX-PG have been reported in children with acute lymphoblastic leukemia (French et al., 2009), even if these studies are still missing in patients with JIA.

Further studies will establish innovative integrated pharmacogenomic and pharmacokinetic features important to identify patients that will respond to methotrexate, a safe and cost-effective agent that remains the first-line therapy of JIA for active and persistent arthritis, in comparison to other more expensive and less safe treatments. Application of pharmacologically guided treatment of JIA will allow rationalization and reduction of costs associated with care, by directing and personalizing the use of methotrexate.

## Conflict of Interest Statement

The authors declare that the research was conducted in the absence of any commercial or financial relationships that could be construed as a potential conflict of interest.
